# Efficacy of GLP-1 analog peptides, semaglutide, tirzepatide, and retatrutide on MC4R deficient obesity and their comparison

**DOI:** 10.1038/s41366-026-02025-2

**Published:** 2026-02-21

**Authors:** Kosuke Hitaka, Takumi Sugawara, Mitsuharu Matsumoto, Yasunori Nio

**Affiliations:** Axcelead Drug Discovery Partners, Inc. Pharmacology Business Unit, Metabolic Syndrome Group, Fujusawa, Kanagawa Japan

**Keywords:** Obesity, Diagnostics

## Abstract

**Introduction:**

Melanocortin 4 receptor (MC4R) is a G-protein-coupled receptor expressed in the hypothalamus, playing a key role in regulating feeding behavior and energy homeostasis. MC4R is integral to the POMC-MC4R and leptin-MC4R pathways, which control food intake and body weight. Mutations in the POMC gene lead to severe early-onset obesity and increased food consumption. Recently, glucagon-like peptide-1 (GLP-1) analogs, including semaglutide, tirzepatide, and retatrutide, have been explored as potential anti-obesity therapies.

**Methods:**

This study aimed to assess and compare the efficacy of these GLP-1 analogs in MC4R knockout (KO) mice, which are deficient in the POMC-MC4R pathway. GLP-1 analogs were administered for 21 days to MC4R KO mice and compared their efficacy.

**Results:**

The percentage of body weight reduction was 19.7 ± 4.1% for semaglutide, 31.6 ± 7.6% for tirzepatide, and 24.1 ± 5.8% for retatrutide. Body composition analysis, including fat and lean mass, was performed using the Echo-MRI system, revealing significant suppression of both fat and lean mass by all three GLP-1 analogs. Furthermore, GLP-1 analogs improved plasma insulin levels, HOMA-IR, cholesterol levels, and markers of liver damage (AST and ALT), as well as reduced liver hypertrophy. While GLP-1 analogs suppressed genes related to fatty acid synthesis, they had no significant effect on inflammation-related gene expressions. Additionally, GLP-1 analogs reduced energy expenditure, with only tirzepatide showing a significant decrease in the respiratory quotient (RQ) in MC4R KO mice.

**Conclusion:**

Our findings demonstrate that all three GLP-1 analogs, semaglutide, tirzepatide, and retatrutide, exhibit significant anti-obesity effects in MC4R KO mice. These results suggest that GLP-1 analogs may provide an effective treatment option for patients with MC4R-POMC pathway deficiencies. Moreover, the efficacy of these drugs in MC4R KO mice aligns with clinical studies, indicating that MC4R KO mice serve as a reliable animal model for obesity research.

## Introduction

Melanocortin 4 receptor (MC4R) is a G-protein-coupled receptor that activates adenylate cyclase. MC4R is expressed in the hypothalamus, and controls feeding behavior and energy homeostasis [1, 2]. MC4R integrates anorectic signals derived from alpha-melanocyte-stimulating hormone (MSH) and orexigenic signals generated by agouti-related peptide (AGRP) [3, 4]. Genetic deficiencies in the POMC-MC4R pathway leads to severe infantile obesity [5, 6]. Prader–Willi syndrome (PWS), a rare genetic disorder, is characterized by hyperphagia. Obesity is a major cause of morbidity and mortality in children and adolescents with this syndrome via deterioration of the POMC-MC4R anorectic pathways [7, 8]. MSH deficiency also causes early phase obesity and increases food intake and body weight [9]. However, there is only one approved drug, setmelanotide for POMC-LEPR deficiency [10], and there is no treatment for other POMC-MC4R pathway-deficient diseases, such as POMC-null, MSH-deficiency, and patients with PWS with obesity. Thus, there is a highly unmet medical need to ameliorate hyperphagia and obesity related to POMC-MC4R pathway deficiency.

Glucagon-like peptide-1 (GLP-1) analogs alter the activity of hypothalamic neurons in the arcuate nucleus, which are directly associated with eating behavior, thereby increasing satiety and reducing energy intake, leading to weight loss [11, 12]. Clinical studies using GLP-1 peptides, such as liraglutide and exenatide, have been conducted in patients with PWS [13–15]. Although there was no significant effect on body weight, BMI, or truncal adiposity, there was a significant decrease in appetite scores and eating behavior [16]. In 2022, a novel GLP-1 analog, semaglutide was introduced as an anti-obesity drug. In clinical trials, liraglutide showed an 8.0% weight loss effect [17], whereas semaglutide showed a 14.9% weight loss effect [18], suggesting that semaglutide has a stronger anti-obesity effect than liraglutide. Liraglutide had a limited effect on the POMC-MC4R pathway deficiency, whereas semaglutide was expected to have a more potent effect. More recently, tirzepatide and retatrutide were studied as novel GLP-1 analogs [19, 20]. These GLP-1 analogs affect glucose-dependent insulinotropic polypeptide receptors and glucagon receptors, in addition to GLP-1R and are expected to exhibit stronger anti-obesity effects than that of semaglutide [21, 22]. The efficacy of semaglutide, tirzepatide, and retatrutide in treating obesity may be promising for patients with POMC-MC4R pathway deficiency. On the contrary, MC4R agonist, setmelanotide, was approved for the rare MC4R-deficiency, Bardet–Biedl syndrome (BBS), with severe obesity in childhood [10, 23]. However, the 5.2% weight loss effect of setmelanotide in patients with BBS aged 12 years and older. The efficacy of setmelanotide may not be sufficient for POMC-MC4R deficient patients because body weight reduction ratio of semaglutide and tirzepatide were 12.4 and 17.8%, respectively in clinical study as compared with that of setmelanotide, 5.2% [[18, 19, 23]]. As MC4R is involved in the regulation of cardiovascular function, there is concern that MC4R activation may affect those functions [24, 25]. Therefore, semaglutide, tirzepatide, and retatrutide are expected to be safer and more effective treatments for MC4R-deficient obesity than setmelanotide. In terms of the anorectic pathways in the brain, the anorectic effects of MC4R agonism are mediated by downstream responses of POMC/CART neurons [26, 27]. The anorectic effects of GLP-1 agonism are reported to result from the suppression of AgRP/NPY neurons and activation of POMC/CART neurons, and these responses occur upstream of MC4R [28, 29]. Therefore, the downstream response blocked by MC4R deficiency could affect the anorexigenic effect of the GLP-1analog, but its effect has not been confirmed yet. Therefore, in this study, we attempted to clarify if GLP-1 analog shows reduction of body weight in POMC-MC4R deficiency using MC4R KO mice. Moreover, we compared which GLP-1 analogs in clinical phase or launched to confirm if MC4R KO mice is to be a good clinically relevant model to evaluate agents for people with obesity.

## Materials and methods

### GLP-1 peptides

GLP-1 peptides, semaglutide (HY-114118, MedChemExpress, Monmouth Junction, NJ, USA), tirzepatide (39748, Cayman Chemical, Ann Arbor, Michigan, USA), and retatrutide (HY-P3506, MedChemExpress) were dissolved in saline (Otsuka Pharma, Tokushima, Japan).

### Animals

For MC4R knockout mice were generated as previously described [30]. In the present study, we used only male mice to minimize variability introduced by the estrous cycle in females, which is known to influence toxicity, body weight, food intake, and metabolic parameters [31–36]. Considering the variability introduced by hormonal fluctuations such as estrogen, only male mice were used to ensure experimental uniformity. Male MC4R KO and C57BL/6 J mice (Jackson Laboratory Japan, Kanagawa, Japan) were fed normal chow (CE-2; CLEA Japan, Tokyo, Japan). Both types of mice were allowed ad libitum access to food and water, and were individually housed under regulated temperature, humidity, and a 12-h light-dark cycle (lights on 7:00–19:00).

### Evaluation of food intake and body weight change

For acclimatization, normal mice and MC4R KO mice were orally administered 0.5% methylcellulose solution daily starting from 7 days before the start of administration. Thirty-five-week-old MC4R mice were randomly divided into four groups with no blinding at one day before drug treatment, vehicle (saline, s.c.), semaglutide (0.5 mg/kg, s.c.), tirzepatide (0.05 mg/kg, s.c.), and retatrutide (0.05 mg/kg, s.c.) based on amounts of food intake from day 2–5 before drug treatment, body weight at day 2 before treatment, and fat mass at one day before treatment. For normal phenotype mice, age-matched C57BL/6 J mice were used. No blinding was performed. Investigators were aware of group allocation during the experiment, but data collection and analyses were conducted using objective measurements to minimize bias. GLP-1 analogs were subcutaneously injected once daily for 21 days. Body weight and food intake were measured 2 and 5 days before dosing, and 1, 2, 3, 5, 7, 10, 12, and 21 days after dosing. One animal was dead just before drug treatment at day 0 in tirzepatide treatment group. Another one animal in tirzepatide treatment group was dead at day 10 after treatment start.

### Evaluation of fat mass and lean mass using Echo-MRI and X-ray CT system

Body components of fat and lean mass were measured using the Echo-MRI system according to the manufacturer’s instructions (Hitachi Aloka Medical Ltd., Tokyo, Japan) at 1 day before dosing and 14 days after dosing without anesthesia. Abdominal visceral and subcutaneous fat mass, and muscle mass were measured using an in vivo X-ray CT system (Latheta LCT-200; Hitachi Aloka Medical) 19 days after dosing. After mice were anesthetized with 3% isoflurane, CT images were acquired using the following parameters: 48 mm axial field of view, and 40 × 96 × 2016 μm voxel size to analyze abdominal visceral and subcutaneous fat mass. Ten cross-sectional images taken from the sacral to lumbar spine were analyzed to quantify abdominal visceral and subcutaneous fat. Fat and muscle mass were analyzed using Latheta software Ver. 3.50.

### Evaluation of energy expenditure and respiratory quotients (RQ)

At day 15 after dosing, the mice were housed individually in the metabolic chamber of the Oxymax system (Columbus Instructions, Columbus, OH, USA) according to the manufacturer’s instructions. At 5:00 pm, mice were administered with the vehicle (saline), semaglutide (0.5 mg/kg, s.c.), tirzepatide (0.05 mg/kg, s.c.), and retatrutide (0.05 mg/kg, s.c.). The metabolic rate and RQ were measured from 7 pm to 1 pm (19:00–7:00, dark phase; 7:00–13:00, light phase).

### Biological sample collection

After 3 weeks of treatment, all mice were anesthetized with isoflurane (3–5%), blood was collected from the abdominal vena cava. Then, all animals were euthanized by bleeding after transection of the abdominal vena cava under anesthesia and the liver, heart, soleus muscle, gastrocnemius muscle, tibialis anterior muscle, extensor digitorum longus muscle, epididymal white adipose tissue, and brown adipose tissue were harvested and immersed in RNAlater solution (Qiagen, Venlo, Netherlands) for qPCR analysis. Blood was centrifuged at 15,000 g. at 4 °C and plasma was collected.

### Analysis of plasma biochemistry

Plasma alanine transaminase (ALT), aspartate aminotransferase (AST), triglyceride, total cholesterol, and glucose levels were measured enzymatically using a Clinical Analyzer 7180 (Hitachi High-Technologies, Tokyo, Japan). Plasma insulin levels were measured using a mouse insulin ELISA kit (Morinaga, Japan). Plasma non-esterified fatty acid (NEFA) levels were measured using Lab Assay NEFA (FUJIFILM Wako Laboratory Chemicals).

### mRNA expression analysis by real-time PCR

Total RNA was isolated from the dissected tissues using the RNeasy Mini kit (Qiagen, CA, USA), followed by reverse transcription using the High Capacity RNA-to-cDNA kit (Thermo Fisher Scientific, Waltham, MA, USA) according to the manufacturer’s instructions. cDNA was amplified using TaqMan Universal Master Mix II (Thermo Fisher Scientific) and ABI7900 (Life Technologies, Tokyo, Japan) according to the manufacturer’s instructions. Commercially available primer-probe sets were used (Applied Biosystems, Waltham, Massachusetts, USA).

Those sets of qRT-PCR probes were as follows: tumor necrosis factor (Tnf, Mm00443260), interleukin 1 beta (Il1b, Mm00434228), interleukin 6 (Il6, Mm00446190), C-C motif chemokine ligand 2 (Ccl2, Mm00441242), stearoyl-CoA desaturase 1 (Scd1, Mm00772290), and fatty acid synthase (Fasn, Mm00662319). The 60S acidic ribosomal protein P0 (Rplp0) (Mm00725448) was used as the endogenous control gene. Relative mRNA expression was calculated by ΔΔCt method.

### Statistical analysis

For animal studies, sample sizes (*n* = 5 per group, 3 in the tirzepatide-treated group) were determined based on prior in-house experience and previous studies using similar mouse models to ensure adequate power to detect biologically relevant effects. No formal statistical sample size estimation was performed. Data are expressed as mean standard deviation. Data involving more than two groups were assessed using Dunnett’s test or one-way analysis of variance, followed by Tukey’s multiple comparisons test. Differences between two groups were assessed using Student’s *t*-test or Aspin–Welch *t* test, followed by Bonferroni’s correction for comparing multiple time points vs. the control group. A *P* value of 0.05 for the Tukey’s multiple comparison test, Dunnett’s test, and Student *t*-test or Aspin–Welch *t*-test was considered to represent a statistically significant difference.

### Ethical approval

All procedures were approved by the Institutional Animal Care and Use Committee (IACUC) of the Shonan Health Innovation Park, iPark Institute Co., Ltd., accredited by AAALAC International, and conducted in accordance with institutional guidelines. All animal experiments were conducted using male mice and approved by the Institutional Animal Care and Use Committee of Shonan Research Center (AU-00040058).

## Results

### Phenotypes of MC4R-deficient mice

MC4R KO mice showed a significant increase in body weight, food intake, fat mass, and lean mass compared with those in normal mice. This is consistent with the characteristics of patients deficient in the POMC-MC4R pathway [5]. In addition, liver injury markers (AST, ALT, and cholesterol) were elevated. Furthermore, MC4R KO mice showed characteristics of insulin resistance, as their glucose levels were comparable to those of normal mice, despite elevated insulin levels (Table 1).

**Table 1:** Comparison of parameters between MC4R KO mice and Normal mice.

	Normal (C57BL/6 J)	MC4R KO
Body weight (g)	34.82 ± 2.15	62.11 ± 4.21**
Food intake (g/day)	3.40 ± 0.31	5.18 ± 0.21**
Fat mass (g)	8.05 ± 1.73	23.85 ± 3.68**
Lean mass (g)	26.95 ± 0.41	38.28 ± 1.26**
AST (U/L)	44.9 ± 14.9	112.3 ± 52.0*
ALT (U/L)	26.8 ± 12.5	143.2 ± 94.4*
Choresterol (mg/dL)	69.9 ± 13.3	152.7 ± 62.3*
Glucose (mg/dL)	255.0 ± 30.8	238.1 ± 35.7
Insulin (ng/mL)	0.94 ± 0.41	11.92 ± 6.31**

Body weight were measured at day 21, 24 hours food intake was measured at day 19, fat/lean mass was measured at day 19, and plasma parameters at day 21 after drug treatment start. Data were compared between vehicle treated normal and vehicle treated MC4R KO mice, mean ± SD, n = 5 each groups.

*ALT* alanine aminotransferase, *AST* aspartate aminotransferase

*p < 0.05, **p < 0.01, vs Normal (Student’s *t*-test).

### Measurement of food intake, body weight, liver, and heart weight

**Fig. 1:**
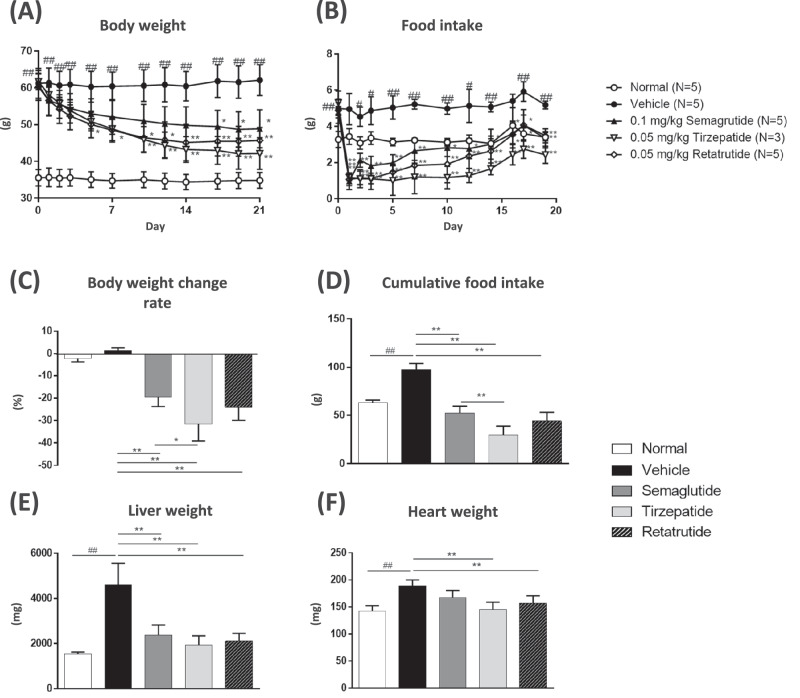
Effects of GLP1 analogs on body weight, food intake, liver and heart weight. **A** Body weight change for 21 days. **B** Food intake change for 19 days. **C** Percentage change in body weight from before to after 21 days of treatment. **D** Cumulative food intake over a 19-day period. Liver and heart weight (**E**–**F**). Data are represented as the mean ± SD from five mice in each group. Data are represented as the mean ± SD from 5 mice per group, 3 mice in the tirzepatide-treated group only. Statistical analysis in (a), (b) were done using Student’s t-test or Tukey test followed by Bonferroni’s correction. Statistical analysis for (c)-(d) was done using Student’s *t*-test or Tukey’s test. #*p* < 0.05, ##*p* < 0.01, vs Normal group by Student’s t-test, *p < 0.05, **p < 0.01, vs Vehicle or Semaglutide group by Tukey’s test.

To evaluate and compare the anti-obesity effect of semaglutide, tirzepatide, and retatrutide in MC4R KO mice, we repeatedly administered vehicle (saline) to normal C57BL/6 J and MC4R KO mice, semaglutide (0.1 mg/kg), tirzepatide (0.05 mg/kg), and retatrutide (0.05 mg/kg) to MC4R KO mice. The mice were subcutaneously administered once daily for 21 days. Body weight and food intake were monitored throughout the treatment period, and liver, heart, and plasma samples were collected 21 days after treatment initiation. The repeated subcutaneous injection of these peptides for 21 days significantly decreased body weight and significantly changed their body weight reduction ratio (semaglutide, 19.7 ± 4.1%; tirzepatide, 31.6% ± 7.6%; and retatrutide, 24.1 ± 5.8%) (Fig. 1A, C). Vehicle-treated MC4R KO mice showed significantly increased food intake compared with that of wild-type mice throughout the study. The repeated administration of all GLP-1 analogs significantly decreased food intake and significantly reduced cumulative food intake amounts during this study (Vehicle; 97.3 ± 6.7 g, semaglutide 52.2 ± 7.1 g, tirzepatide 29.7 ± 9.0 g, retatrutide 43.9 ± 9.0 g) (Fig. 1B, D). Effect of semaglutide was weaker than that of tirzepatide. All three GLP-1 analogs significantly ameliorated liver and heart hypertrophy (Fig. 1E, F).

### Evaluation of fat mass and lean mass using Echo-MRI and X-ray CT system

**Fig. 2:**
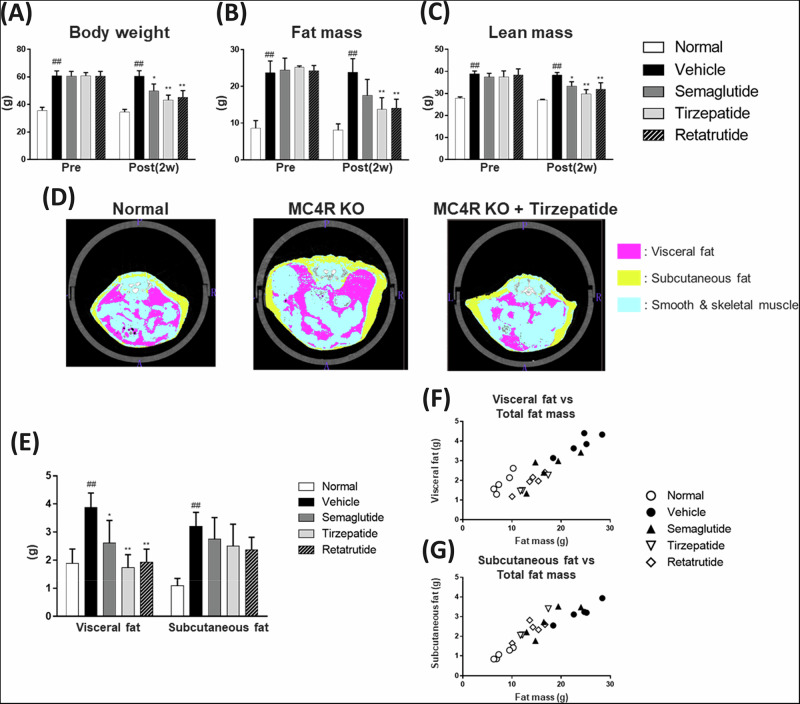
Changes in body weight and body composition after treatment with GLP-1 analogs. Body weight (**A**), Fat and lean mass (**B**–**C**). Scout image of mice whole body scanned by X-ray CT. Cross-sectional image of the peri-abdominal area imaged by X-ray CT. **D** Quantitative evaluation value of visceral and subcutaneous fat from segmental CT images **E**. Correlation of visceral fat mass (**F**) and subcutaneous fat mass (**G**) with total fat mass were calculated. Data are represented as the mean ± SD from 5 mice per group, 3 mice in the tirzepatide-treated group only. Statistical analysis was done using Student’s *t*-test or Tukey’s test. #*p* < 0.05, ##*p* < 0.01, vs Normal group by Student’s t-test, *p < 0.05, **p < 0.01, vs Vehicle group by Tukey’s test.

Consistent with the significant increase in body weight, vehicle-treated MC4R KO mice showed a significant increase in fat and lean masses. After 2 weeks, repeated subcutaneous injections of these peptides significantly decreased body weight and fat mass. Similar to a previous clinical study [18], these peptides significantly reduced lean mass, indicating that these GLP-1 analog peptides suppressed both fat and lean mass (Fig. 2A–C). To analyze body composition in detail, we used an X-ray CT system and evaluated the amounts of visceral and subcutaneous white adipose tissue, skeletal muscle, and smooth muscle (Fig. 2D). As the result, GLP-1 analogs significantly reduced visceral fat and showed a trend to reduce subcutaneous fat. (Fig. 2E). Visceral and subcutaneous fat volumes showed changes that reflected the fat mass (visceral fat vs. fat mass: R^2^ = 0.79; subcutaneous fat vs. fat mass: R^2^ = 0.84) (Fig. 2F–G).

### Evaluation of energy expenditure and RQ

**Fig. 3:**
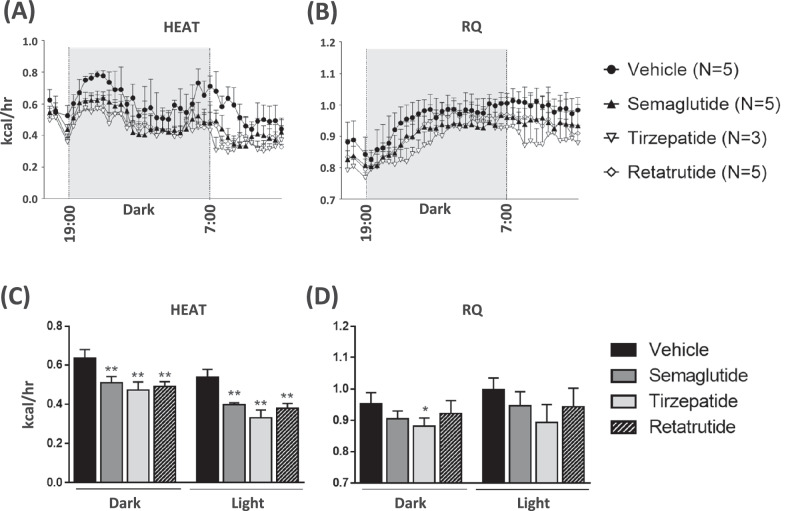
Measurement of energy expenditure and respiratory quotient in mice after treatment with GLP-1 analogs. Time course of the metabolic rates was monitored continuously for 18 h by using an Oxymax system. The mean of energy expenditure, HEAT (**A**) and respiratory quotient, RQ (**B**) per light and dark period. Average HEAT (**C**) and RQ (**D**) in dark and light phase were calculated. Data are represented as the mean ± SD from 5 mice per group, 3 mice in the tirzepatide-treated group only. Statistical analysis was done using Tukey’s test. *p < 0.05, **p < 0.01, vs Vehicle group by Tukey test.

To evaluate the metabolic changes after GLP-1 analog treatment, energy expenditure, and RQ were measured. Repeated administration of the three GLP-1 analogs significantly reduced the metabolic rate in both the light and dark phases (Fig. 3A, C). This is the first time-course study to evaluate and compare three clinical GLP-1 analogs in MC4R KO mice. Considering these time-course study results, the vehicle treatment group showed increased energy expenditure just before and after the light and dark cycles, indicating that these time points were feeding times. In contrast to the vehicle-treated group, the GLP-1 analog-treated group did not show an increase in energy expenditure at these time points (Fig. 3A, C). In addition, only tirzepatide significantly reduced RQ (Fig. 3B, D).

### Evaluation of muscle amounts

Clinical trials of semaglutide have reported a decrease in muscle mass, which increases the risk of sarcopenia, particularly in skeletal muscle [37]. In our study, all three GLP-1 analogs reduced the lean mass (Fig. 2B). To further evaluate the effects of GLP-1 analogs on muscle mass, four types of skeletal muscles (soleus, gastrocnemius, tibialis anterior, and extensor digitorum longus) were independently measured after 3 weeks of treatment. Similar to the clinical data, all three GLP-1 analogs tended to reduce skeletal muscle mass. Retatrutide significantly reduced the soleus muscle weight (Table 2).

**Table 2:** Comparison of various muscle weights of the right thigh after GLP-1 treatment.

	Vehicle	Semaglutide	Tirzepatide	Retatrutide
Soleus muscle (mg)	13.36 ± 0.43	11.70 ± 0.94	11.91 ± 1.91	11.10 ± 1.15*
Gastrocnemius muscle (mg)	173.50 ± 8.18	154.68 ± 17.11	152.67 ± 17.41	157.76 ± 13.08
Tibialis anterior muscle (mg)	59.78 ± 4.18	55.23 ± 3.71	55.62 ± 5.50	55.42 ± 5.58
Extensor digitorum longus (mg)	12.02 ± 4.44	12.01 ± 2.84	12.15 ± 1.43	12.98 ± 2.37
Total (mg)	258.67 ± 12.16	233.62 ± 22.60	232.35 ± 25.80	237.26 ± 21.59

Measured soleus, gastrocnemius, tibialis anterior, extensor digitorum longus and their total in the right thigh. Data are represented as the mean ± SD from 5 mice per groups, 3 mice, tirzepatide treated group. Statistical analysis was done using Dunnett’s test.

*p < 0.05, vs Vehicle group.

### Measurement of plasma parameters

**Fig. 4:**
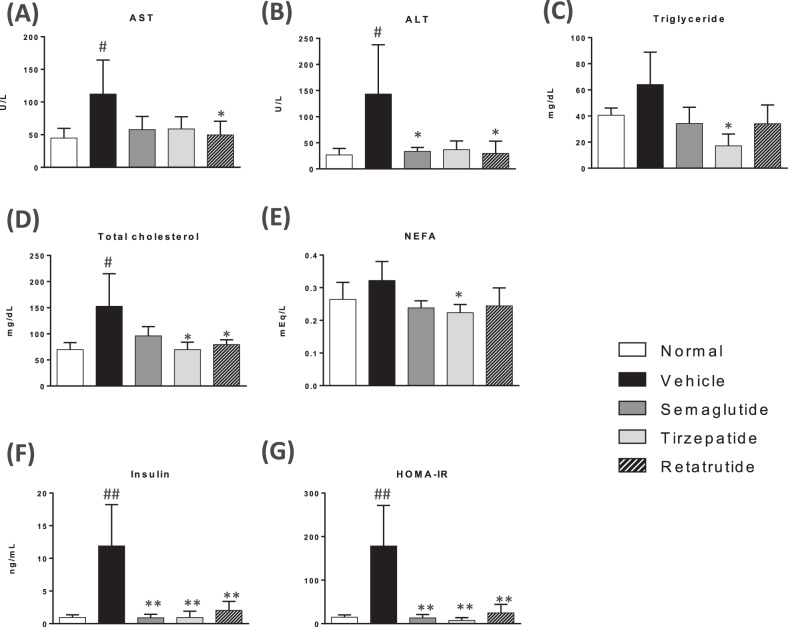
Effect of GLP-1 analogs on liver injury markers and plasma parameters. Plasma AST (**A**), ALT (**B**), triglyceride (TG) (**C**), total cholesterol (TC) (**D**), nonesterified fatty acid (NEFA) (**E**), insulin levels (**F**) and HOMA-IR (**G**). Plasma ALT and AST levels for 7 weeks. **B** Plasma TIMP-1 concentration. **C** Time course of rate of plasma LDL-C concentration from the values before treatment. **D** Plasma total T3 concentration. **E** Plasma total cholesterol and triglyceride concentration. Data are represented as the mean ± SD from 5 mice per group, 3 mice in the tirzepatide-treated group only. Statistical analysis was done using Tukey’s test. *p < 0.05, **p < 0.01, vs Vehicle group by Tukey test.

Similar to the clinical data from patients with obesity [38], MC4R KO mice showed significantly increased levels of markers of liver injury, including AST, ALT, TC, and TG (Fig. 4A–D). Moreover, the MC4R KO mice also increased plasma insulin levels and HOMA-IR values, indicating insulin resistance (Fig. 4G, H). All GLP-1 analogs significantly or slightly reduced plasma AST, ALT, TG, TC, insulin, and HOMA-IR levels (Fig. 4A–D, G, H). Among them, tirzepatide significantly decreased TG, cholesterol, and NEFA levels (Fig. 4C–E).

### Gene expression analysis

**Fig. 5:**
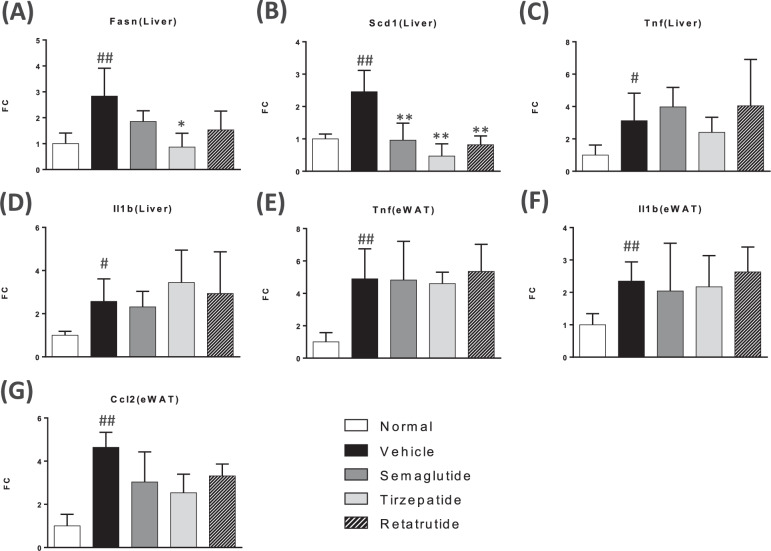
Gene expression analysis of GLP-1 analogs on fatty acid synthesis and pro-inflammatory factors in liver and white adipose tissue. mRNA expression of Fas (**A**), Scd-1 (**B**), Tnf-alpha (**C**), Il1-beta (**D**) in liver, Tnf-alpha (**E**), Il1-beta (**F**), and Ccl2 (**G**) were shown. Data are represented as the mean ± SD from 5 mice per group, 3 mice in the tirzepatide-treated group only. Statistical analysis was done using Tukey’s test. *p < 0.05, **p < 0.01, vs Vehicle group by Tukey test.

MC4R KO mice showed a significant increase in fatty acid synthesis-related genes, such as FASN and SCD-1 (Fig. 5A, B), indicating steatosis. In terms of inflammation, MC4R KO mice showed pro-inflammatory gene expression, such as TNF-alpha and IL1-beta in liver and white adipose tissue (WAT), indicating inflammation are activated in liver and WAT (Fig. 5C–G). All GLP-1 analogs suppressed *Scd1* and *Fasn* gene expression in the liver, which are involved in lipid synthesis, was downregulated in all GLP-1 analog treatment groups (Fig. 5A, B). Tirzepatide showed stronger suppressive effects on gene expression than those with semaglutide and retatrutide. In contrast, none of the GLP-1 analogs showed any significant suppressive effects on pro-inflammatory gene expression (Fig. 5C–G).

## Discussion

It was unclear whether GLP-1 analogs are effective in models lacking the MC4R pathway. Therefore, in this study, we conducted an anti-obesity evaluation using MC4R-deficient mice to clarify whether GLP-1 analogs show anti-obesity effects even in MC4R pathway-deficient models and to determine which of the recently launched GLP-1 analogs is most effective. Additionally, the effects of GLP-1 analogs on muscle mass were examined, as the impact on muscle mass is a significant concern in weight management. While the weight-loss effect of GLP-1 analogs is largely attributed to potent suppression of food intake, this reduction in food intake affects not only fat mass but also muscle mass [39]. Chronic suppression of food intake could lead to muscle loss, potentially resulting in sarcopenia [40]. This is a critical issue, as a decrease in muscle mass leads to a reduction in basal metabolic rate [41, 42], which could hinder the long-term benefits of obesity treatment by impairing the overall metabolic profile. In some cases, the discontinuation of GLP-1 therapy can result in weight regain due to a rebound effect [43, 44]. These concerns highlight the necessity for ongoing treatment and monitoring during GLP-1 therapy to ensure sustainable weight loss while minimizing adverse outcomes, such as sarcopenia. Given the importance of muscle mass in maintaining metabolic health, we examined the impact of GLP-1 analogs on both muscle mass and energy expenditure in this study. To investigate the metabolic effects more comprehensively, we used the Oxymax system to measure energy expenditure and metabolic changes and the Echo-MRI system as well as X-ray CT to assess muscle mass changes. The dose of semaglutide used in this study was based on the highest clinically applicable dose of 2.4 mg, as determined by clinical trial data [45]. Tirzepatide was administered at a dose assumed to be below the lowest clinically applicable dose of 5 mg, based on C_max_ [46], and the same dose was used for retatrutide. All three GLP-1 analogs showed marked suppression of food intake and rapid weight loss effects immediately after administration (Fig. 1A–D). These effects were particularly strong during the initial phase of treatment, and sustained suppression of food intake and weight loss continued throughout the three-week evaluation period. The sustained nature of the weight loss observed in this study would be in line with clinical findings that show greater weight loss with tirzepatide compared to semaglutide [47]. These findings indicate that GLP-1 analogs exert significant anti-obesity effects even when the MC4R signaling pathway is disrupted. Importantly, GLP-1 analogs appear to exert their anti-obesity effects through central pathways that do not involve MC4R, as well as via peripheral mechanisms involving the vagus nerve [48, 49]. This observation is particularly important because it demonstrates that these drugs can remain effective even in the absence of the MC4R pathway, which is traditionally thought to play a key role in regulating energy balance and appetite. This finding opens up new therapeutic avenues for patients with MC4R mutations or dysfunction, where conventional weight loss strategies may not be effective. Energy expenditure measurements revealed a reduction in energy consumption for all three GLP-1 analogs (Fig. 3A, C). Tirzepatide and retatrutide, which have dual agonist activities targeting both GLP-1 receptor and GIP receptor (GIPR) or GLP-1 receptor and glucagon receptor (GCGR), respectively, have been shown to improve energy expenditure and metabolic flexibility in other studies [50, 51]. However, we did not observe these effects in the present study, despite administering doses that are comparable to those used in clinical trials. This discrepancy could be due to the duration of treatment, as our evaluation period was three weeks, whereas previous studies have assessed the effects over shorter time frames. Furthermore, changes in body composition, particularly with regard to muscle mass, may have influenced the energy expenditure findings, highlighting the need for longer-term studies to fully assess the effects of GLP-1 analogs on metabolism. Previous reports have also shown that GLP-1 treatment can reduce locomotor activity [52, 53]. Therefore, the observed decrease in energy expenditure in our study is likely attributable not only to reduced food intake but also to reduced locomotor activity induced by GLP-1 analogs. The impact of GLP-1 analogs on muscle mass is of particular concern, as a reduction in muscle mass can lead to sarcopenia, which negatively affects metabolic health and increases the risk of frailty in the elderly. Our study demonstrated that GLP-1 analogs reduced lean mass in MC4R KO mice, which is in line with previous reports indicating that GLP-1 treatment can cause muscle loss [37]. Interestingly, while the weights of four different skeletal muscles were measured, no significant changes were observed, although a trend toward decreased muscle mass was noted. We evaluated the differences on the muscle mass change among these GLP-1 analogs, there is no significant differences among them. Although all three GLP-1 analogs tended to suppress muscle mass as similar manner, tirzepatide showed the biggest body weight reduction similar with clinical study. Therefore, tirzepatide seemed to be superior rather than semaglutide and retatrutide considering this study. To avoid muscle mass by GLP-1 analogs, combination therapy of muscle mass inducer such as Bimagrumab (Anti-ACVR2B Antibody) [54] with GLP-1 analogs would be a good treatment regimen to suppress muscle mass reduction leads to sarcopenia. Among the GLP-1 analogs, tirzepatide showed the most pronounced decrease in respiratory quotient (RQ) (Fig. 3B, D), which reflects a shift in metabolism towards increased fat oxidation. This is consistent with the robust weight loss and reduction in food intake observed with tirzepatide (Fig. 1A–D). While GLP-1R is known to be expressed in the central nervous system [55], the effectiveness of GLP-1 analogs in patients with MC4R-POMC pathway deficiency has not been well established. Our results are the first to suggest that GLP-1 analogs are effective in patients with MC4R pathway deficiency, as evidenced by the observed improvements in body weight, food intake, and metabolic parameters. These findings have important clinical implications, as they suggest that GLP-1 analogs could be a viable treatment option for patients with MSH, PSW, or other forms of obesity resulting from MC4R deficiencies. Analysis of blood parameters confirmed improvements in key markers of glucose and lipid metabolism, such as blood lipid levels, cholesterol, liver damage markers, and HOMA-IR. Genetic analysis revealed downregulation of genes associated with lipid synthesis, such as FAS and SCD1, in the liver. In contrast, inflammation-related genes were upregulated in the liver and adipose tissue, although there was no significant effect with the GLP-1 analog treatments. This suggests that the improvements in metabolic parameters were primarily driven by the modulation of lipid and glucose metabolism, rather than the suppression of inflammation. In addition to the metabolic and phenotypic parameters reported in the present study, previous research has demonstrated that melanocortin-4 receptor (MC4R) deficiency is associated with profound alterations in multiple hypothalamic neuropeptides and peripheral hormones involved in energy balance regulation. For example, MC4R knockout mice exhibit significantly increased hypothalamic expression of neuropeptide Y (NPY) and agouti-related protein (AgRP), while pro-opiomelanocortin (POMC) expression is decreased compared with wild-type controls [56]. Circulating ghrelin levels are reduced in female MC4R knockout mice, and these animals show decreased sensitivity to ghrelin’s effects on food intake and growth hormone secretion [57]. Furthermore, MC4R-deficient mice develop severe obesity accompanied by markedly elevated plasma leptin concentrations and leptin resistance, failing to respond to leptin’s anorectic and cardiovascular effects [58, 59]. Complementary findings in dietary obesity models have also been reported, where AgRP levels were elevated under hyperphagic conditions and reduced with food restriction, while α-MSH and POMC concentrations remained unchanged [60]. Together, these studies underscore the profound neuroendocrine adaptations associated with MC4R dysfunction.

To our knowledge, however, there are currently no reports describing how GLP-1 receptor agonist treatment modulates the expression of BDNF, leptin, ghrelin, NPY, α-MSH, or AgRP in the context of MC4R deficiency. Our study was not designed to address this question directly, and we therefore did not include such analyses. Nonetheless, considering the clinical relevance of GLP-1 therapies and their potential impact on energy balance beyond food intake and body weight, future studies should investigate whether GLP-1 agonists influence hypothalamic neuropeptide expression or signaling cascades in MC4R-deficient models. Such analyses may provide important mechanistic insight into the intersection between GLP-1 and melanocortin pathways and could help to explain differential therapeutic responses in patients with distinct genetic backgrounds. Heart failure is a common complication of obesity [61, 62], and clinical trials have shown that GLP-1 analogs, particularly tirzepatide, can improve cardiac function and reduce the risk of heart failure in people with obesity [63]. In our study, we observed a modest improvement or slight reduction in cardiac hypertrophy in MC4R KO mice treated with GLP-1 analogs, which further supports the notion that these drugs have potential cardiovascular benefits. However, the clinical relevance of these findings in the context of MC4R deficiency remains to be fully explored in larger animal models and human trials. Although this study provides valuable insights into the effects of GLP-1 analogs on MC4R deficiency pathology, there are several limitations that must be addressed in future studies. First, although we initially planned to include five mice per group, one animal in the tirzepatide-treated group died just before drug administration (day 0), and an additional mouse died on day 10 after treatment initiation. Consequently, the tirzepatide group was reduced to three animals by the end of the study. This reduction in sample size should be taken into consideration when interpreting the tirzepatide-related outcomes. We acknowledge that only male mice were used in this study to avoid variability from the female estrous cycle, and therefore sex-specific differences could not be evaluated. The three-week observational period may not be sufficient to fully assess the long-term effects of GLP-1 analogs on muscle mass, metabolic health, and cardiovascular function. Another limitation is that we did not directly measure body length of wild-type and MC4R KO mice. Based on visual inspection during the experimental period (35–38 weeks old), MC4R KO mice appeared to have a longer body length compared to wild-type controls, which is consistent with the findings reported by Braun et al. [64]. This limitation should be considered when interpreting the metabolic rate data, as body size differences may affect energy expenditure normalization. It is possible that more pronounced changes in muscle mass or metabolic improvements could be observed with longer treatment periods. Also, the lack of a comprehensive analysis of muscle mass across multiple muscle groups and tissues may have limited our ability to fully assess the impact of GLP-1 analogs on muscle health. This study included genetic analysis, but did not measure protein expression levels, which limits the interpretation of the results. Regarding the effect of the GLP-1 agonist, since it suppressed the expression of liver lipid synthesis genes FAS and SCD1, and consequently reduced liver triglyceride levels, its effect could be confirmed without measuring protein levels. However, for inflammatory factors, since there were no changes in gene expression due to the drug treatment, protein levels were not measured. Nevertheless, we cannot definitively say that there were no changes in protein levels of inflammatory factors. In conclusion, our results indicate that GLP-1 analogs have significant anti-obesity effects in MC4R and POMC pathway-deficient models, providing evidence of their potential effectiveness in patients with these rare genetic conditions. The suppression of energy expenditure and muscle mass loss observed with reduced dietary intake underscores the need for careful management of these therapies in clinical practice. This study provides the first demonstration that GLP-1 analogs can be effective in treating obesity associated with MC4R deficiency in mice, offering new therapeutic possibilities for patients lacking the POMC-MC4R pathway.

## Data Availability

The raw data generated and/or analyzed during the current study are available from the corresponding author upon reasonable request.
